# Fear memory regulation by the cAMP signaling pathway as an index of reexperiencing symptoms in posttraumatic stress disorder

**DOI:** 10.1038/s41380-024-02453-4

**Published:** 2024-02-27

**Authors:** Hiroaki Hori, Hotaka Fukushima, Taikai Nagayoshi, Rie Ishikawa, Min Zhuo, Fuyuko Yoshida, Hiroshi Kunugi, Kenichi Okamoto, Yoshiharu Kim, Satoshi Kida

**Affiliations:** 1 Department of Behavioral Medicine, National Institute of Mental Health, National Center of Neurology and Psychiatry, Tokyo, 187-8553 Japan; 2 Department of Bioscience, Graduate School of Life Sciences, Tokyo University of Agriculture, Tokyo, 156-8502 Japan; 3 Graduate School of Agriculture and Life Sciences, The University of Tokyo, Tokyo, 113-8657 Japan; 4 Department of Physiology, Faculty of Medicine, University of Toronto, 1 King’s College Circle, Toronto, ON Canada; 5 Department of Mental Disorder Research, National Institute of Neuroscience, National Center of Neurology and Psychiatry, Tokyo, 187-8502 Japan; 6 Department of Psychiatry, Teikyo University School of Medicine, Tokyo, 173-8605 Japan; 7 Lunenfeld-Tanenbaum Research Institute, Mount Sinai Hospital, 600 University Avenue, Toronto, ON Canada; 8 Department of Molecular Genetics, Faculty of Medicine, University of Toronto, 1 King’s College Circle, Toronto, ON Canada

**Keywords:** Neuroscience, Molecular biology

## Abstract

Posttraumatic stress disorder (PTSD) is a psychiatric disorder associated with traumatic memory, yet its etiology remains unclear. Reexperiencing symptoms are specific to PTSD compared to other anxiety-related disorders. Importantly, reexperiencing can be mimicked by retrieval-related events of fear memory in animal models of traumatic memory. Recent studies revealed candidate PTSD-associated genes that were related to the cyclic adenosine monophosphate (cAMP) signaling pathway. Here, we demonstrate the tight linkage between facilitated cAMP signaling and PTSD by analyzing loss- and gain-of-cAMP signaling effects on fear memory in mice and the transcriptomes of fear memory-activated mice and female PTSD patients with reexperiencing symptoms. Pharmacological and optogenetic upregulation or downregulation of cAMP signaling transduction enhanced or impaired, respectively, the retrieval and subsequent maintenance of fear memory in mice. In line with these observations, integrative mouse and human transcriptome analysis revealed the reduced mRNA expression of phosphodiesterase 4B (PDE4B), an enzyme that degrades cAMP, in the peripheral blood of PTSD patients showing more severe reexperiencing symptoms and the mouse hippocampus after fear memory retrieval. Importantly, more severe reexperiencing symptoms and lower *PDE4B* mRNA levels were correlated with decreased DNA methylation of a locus within *PDE4B*, suggesting the involvement of methylation in the mechanism of PTSD. These findings raise the possibility that the facilitation of cAMP signaling mediating the downregulation of PDE4B expression enhances traumatic memory, thereby playing a key role in the reexperiencing symptoms of PTSD patients as a functional index of these symptoms.

## Introduction

Posttraumatic stress disorder (PTSD) is a serious psychiatric condition that can develop after a major traumatic event, with an estimated lifetime prevalence of approximately 3.9% worldwide [1]. Patients with PTSD exhibit a variety of psychological and behavioral symptoms, including reexperiencing, avoidance, and hyperarousal. Reexperiencing refers to the involuntary retrieval of traumatic memories, such as flashbacks, nightmares, and intrusive thoughts. While avoidance and hyperarousal are common to many anxiety-related disorders, reexperiencing is largely unique to PTSD and is recognized widely as a core feature of this disorder [2]. Since reexperiencing can be understood as a persistent conditioned response, i.e., a learned response to a previously neutral stimulus [3], the learning and memory processes involved in fear memory regulation are postulated to underlie reexperiencing symptoms [4, 5].

The development of PTSD depends on environmental factors such as traumatic experiences. However, there is growing evidence that genetic factors also play an important role, indicating that PTSD is caused by a combination of environmental and genetic factors. Pituitary adenylate cyclase-activating polypeptide (PACAP) in peripheral blood is associated with PTSD symptoms in females, while a single nucleotide polymorphism in the PAC1 receptor gene (*ADCYAP1R1*) is associated with PTSD symptoms in females, and shows fear- and estrogen-induced expression [6]. Interestingly, a genome-wide association study identified corticotropin-releasing hormone receptor 1 gene (*CRHR1*) as relevant to intrusive reexperiencing in PTSD [7]. Since PACAP, PAC1, and CRHR1 activate the cyclic adenosine monophosphate (cAMP) signaling pathway, these findings suggest that this pathway may influence the etiology of PTSD. However, the mechanism by which genetic factors contribute to the pathogenesis of PTSD has not been elucidated.

Fear memory is generated through traumatic experiences. In experimental animals, Pavlovian fear conditioning and inhibitory avoidance (IA) tasks, both of which generate fear memory, have been used widely as models of PTSD. Fear memory is initially labile up to several hours after a fear experience and then stabilized through gene expression-dependent memory consolidation [8–12]. Importantly, memory retrieval is not a passive process; consolidated memory becomes labile again when it is retrieved and then maintained or enhanced through gene expression-dependent reconsolidation (re-stabilization) [13–16]. Conditioned fear memory has been observed in many animal species from insects to humans [17–21]; therefore, the mechanisms for fear memory regulation may be similar between humans and other animals.

Reexperiencing may involve the dysregulation of memory processes related to the retrieval, maintenance, and reconsolidation of traumatic memory. Rodent studies including ours have suggested that retrieval and the subsequent memory processes are facilitated by activation of the cAMP signaling pathway [22–24]. Therefore, it is possible that the state of cAMP signaling may correlate with the state of traumatic memory.

In the present study, we first examined the relationship between the state of cAMP signaling and the retrieval and subsequent fate of fear memory using behavioral pharmacology and optogenetic experiments in mice. To identify the common molecular signatures underlying trauma-related reexperiencing symptoms and fear memory retrieval, we then integratively analyzed human blood transcriptomes in relation to reexperiencing symptoms and mouse hippocampal transcriptomes following the retrieval of fear memory. The key gene identified was investigated further by gene co-expression analysis and DNA methylation analysis in humans. Moreover, the gene expression observed in the mouse hippocampus was confirmed using mouse blood samples.

## Materials and methods

Detailed methods are provided in Supplementary Information.

### Mouse study

#### Mice

Male C57BL/6N mice were obtained from Charles River (Yokohama, Japan). The mice were housed in cages of 5 or 6, maintained on a 12-h light/dark cycle, and allowed access to food and water *ad libitum*. The mice were at least 8 weeks of age when tested. Testing was performed during the light phase of the cycle. All experiments were conducted blind to the treatment condition of the mice (*n* = 225 in total).

#### Drug injection and behavioral tests

The detailed method for the systemic injection of rolipram (ROL), a phosphodiesterase 4 (PDE4) inhibitor [25], and NB001, an adenylyl cyclase 1 inhibitor [26], is shown in Supplementary Information. ROL was dissolved in dimethyl sulfoxide and diluted with distilled water for systemic injection. NB001 was dissolved in saline for systemic injection. Behavioral experiments, including the contextual fear conditioning test [23, 27–30] and IA test [31–34] were performed as described previously (for details, see Supplementary Information). Behavioral experiments were performed in a blinded manner.

#### Optogenetic experiments

The detailed methods for the viruses, virus injection, optical fiber implantation, and optogenetic manipulation of cAMP levels in the dorsal hippocampus are shown in Supplementary Information. To examine the effects of increased or decreased cAMP levels on fear memory, mice received a micro-infusion of an AAV vector expressing a photoactivatable adenylyl cyclase (bPAC) (AAV9-CaMKII-mGFP-bPAC, titer: 1.21 × 10^13^ vg/mL) or a light-activated phosphodiesterase (LAPD) (AAV9-CK0.4-LAPD-GFP, titer: 3.16 × 10^13^ vg/mL) under the control of the CaMKII promoter into the dorsal hippocampus, respectively [35].

#### RNA analysis

The detailed methods for RNA extraction, quantitative reverse transcription PCR (qRT-PCR), and RNA-sequencing (RNA-seq) are shown in Supplementary Information. Total RNA was isolated from the mouse dorsal hippocampus using an RNeasy Mini Kit (Qiagen) according to the manufacturer’s protocol for animal tissues [23, 36]. To analyze peripheral blood mRNA, the mice were anesthetized before blood collection by cardiac puncture [37]. Total RNA from peripheral blood was isolated using RNAprotect Animal Blood Tubes (Qiagen) and an RNeasy Protect Animal Blood Kit (Qiagen) according to the manufacturer’s instructions.

#### Statistical analysis

One-way analysis of variance (ANOVA) followed by a *post hoc* Newman–Keuls test and two-way ANOVA followed by *post hoc* Bonferroni’s comparisons were used to analyze the effects of drug, time, and group. A paired *t*-test was used to analyze the differences in crossover latency within each group between two sessions (re-exposure vs. test). Student’s *t*-test was used to analyze differences in mRNA expression levels.

### Human study

#### Participants

This study was approved by the ethics committee of each institute involved (including National Center of Neurology and Psychiatry, Tokyo Women’s Medical University, and Nagoya City University), and written informed consent was obtained from all participants.

Details of participant recruitment are described in our previous paper [38]. Briefly, the sample comprised 32 civilian patients with PTSD and 16 healthy controls. This sample size was determined by referring to previous blood-based transcriptome studies in PTSD [39–41]. All participants were Japanese women, with ages ranging from 21 to 59 years. Patients with PTSD were consecutively recruited at the institutes and their affiliated hospitals/clinics located in metropolitan areas in Japan. All the patients had been diagnosed as having PTSD by their attending clinicians. The experience of traumatic events and diagnosis of PTSD were confirmed by the Japanese version [42] of the Posttraumatic Diagnostic Scale (PDS) [43], a well-established self-administered scale. For PTSD diagnosis, the PDS shows a high concordance rate (i.e., 95.1%, *κ* = 0.90) [42] with the Clinician-Administered PTSD Scale [44], a gold-standard structured interview. Patients with comorbid schizophrenia and those with marked manic episodes of bipolar disorder were excluded from the study. PTSD symptom severity of the patients was assessed using the validated Japanese version [45] of the Impact of Event Scale-Revised (IES-R) [46], a 22-item self-report questionnaire measuring the three core PTSD symptom clusters: reexperiencing (intrusion), avoidance, and hyperarousal. In addition, healthy controls were recruited mostly through advertisements in free local magazines and on our website. All the healthy individuals were interviewed by a board-certified psychiatrist in order to ascertain that they were psychiatrically healthy and were not taking any psychotropic medications. Anxiety symptoms and depression symptoms were also assessed with validated self-reported questionnaires in both patients and controls.

#### Blood sampling

For each participant, blood sampling was performed before lunch (11:30–12:30). The collected blood was used for RNA and DNA analyses.

#### Transcriptome analysis

Transcriptome measurements were performed using Agilent SurePrint G3 v3 human GE 8 × 60 K microarrays (Agilent Technologies). The raw signal values were thresholded to 1.0, and log base 2-transformation was performed. The 75^th^ percentile shift normalization and baseline transformation with the median of all samples were then conducted. The normalized data were used for all statistical analyses. Microarray data have been deposited to the Gene Expression Omnibus database repository with the dataset identifier GSE199841.

#### DNA methylation analysis

DNA methylation levels were measured with an Infinium MethylationEPIC BeadChip (Illumina). The BeadChip was scanned on the Illumina iScan system, and background subtraction and normalization to internal controls were performed with the GenomeStudio Methylation Module software (Illumina). For the genome-wide DNA methylome data, our analysis focused on CpG sites in the target gene, i.e., phosphodiesterase 4B (*PDE4B*).

#### Data analysis

Correlations between gene expression levels and other variables were calculated by Pearson’s correlation coefficient (*r*) and further confirmed by Spearman’s rank order correlation (ρ) where necessary. Student’s *t*-test was used to compare gene expression levels between patients and controls. Statistical significance was set at two-tailed *p* < 0.05 unless otherwise specified.

## Results

### Retrieval and maintenance of fear memory correlate with cAMP levels

**Fig. 1:**
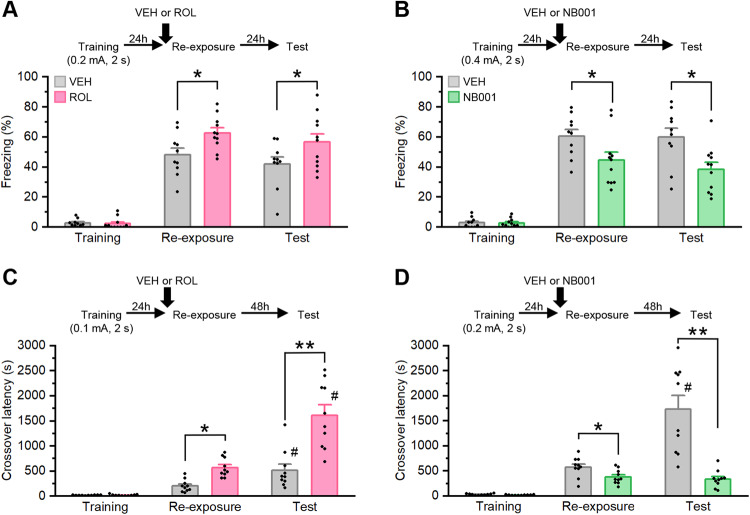
Effects of the systemic injection of rolipram or NB001 on fear memory retrieval and fate. Effects of the systemic injection of rolipram (ROL) (**A**) or NB001 (**B**) on contextual fear memory. **A** Vehicle (VEH), *n* = 10; ROL, *n* = 11. (**B**), VEH, *n* = 10; NB001, *n* = 11. Effects of the systemic injection of ROL (**C**) or NB001 (**D**) on IA memory. **C** VEH, *n* = 10; ROL, *n* = 10. (**D**), VEH, *n* = 10; NB001, *n* = 10. *p < 0.05, **p < 0.01, *post hoc* Bonferroni’s test; #*p* < 0.05, paired *t*-test. Error bars indicate SEM.

We first examined the effect of cAMP levels on the retrieval and subsequent fate of hippocampus-dependent contextual fear memory using pharmacology. To examine the effects of increased cAMP levels on contextual fear memory, the mice received a systemic injection of ROL, a PDE4 inhibitor [25], or vehicle (VEH) before memory retrieval. The mice were trained with a single footshock (0.2 mA, 2 s; training) and then re-exposed to the training context for 3 (re-exposure) and 5 (test) min every 24 h. The mice were systemically injected with ROL (0.1 mg/kg b.w.) or VEH at 30 min before re-exposure. Two-way ANOVA revealed significant effects of drug (ROL vs. VEH: *F*_(1,38)_ = 10.191, *p* < 0.05), but not time (re-exposure vs. test: *F*_(1,38)_ = 1.775, *p* > 0.05) or time × drug interaction (*F*_(1,38)_ = 0.000, *p* > 0.05) (Fig. 1A). The ROL group froze significantly more than the VEH group at re-exposure and test (*ps* < 0.05; *post hoc* Bonferroni’s test). These results suggest that ROL injection enhances the retrieval and subsequent maintenance of contextual fear memory. Thus, our observations suggest that the upregulation of cAMP levels strengthens contextual fear memory.

To examine the effects of decreased cAMP levels, we used NB001, an adenylyl cyclase 1 inhibitor [26]. We performed a similar experiment as above, except that the mice were trained with a footshock (0.4 mA, 2 s) and systemically injected with NB001 (30 mg/kg b.w.) or VEH twice every hour from 2 h before re-exposure. The NB001 group froze significantly less than the VEH group at re-exposure and test (two-way ANOVA: drug, *F*_(1,38)_ = 13.317, *p* < 0.05; time, *F*_(1,38)_ = 0.43, *p* > 0.05; and time × drug interaction, *F*_(1,38)_ = 0.305, *p* > 0.05; *post hoc* Bonferroni’s test, *ps* < 0.05; Fig. 1B). These results indicate that NB001 impairs the retrieval and subsequent expression of contextual fear memory, suggesting that the downregulation of cAMP levels disrupts contextual fear memory.

We examined the effects of pharmacological activation (ROL) and inactivation (NB001) on IA memory, another type of hippocampus-dependent fear memory. The mice were first placed in the light compartment. At 5 s after they entered the dark compartment, a brief electrical footshock (0.1 mA, 2 s) was delivered (training). The mice were re-exposed to the light compartment at 24 h after training and their crossover latency to enter the dark compartment was assessed (re-exposure). The mice were returned to their home cages immediately after they entered the dark compartment from the light compartment. Prior to re-exposure, the mice received a systemic injection of VEH or ROL. At 48 h later, crossover latency was assessed (test). The ROL group showed significantly longer crossover latency at re-exposure and test than the VEH group (two-way ANOVA: drug, *F*_(1,36)_ = 30.872, *p* < 0.05; time, *F*_(1,36)_ = 24.355, *p* < 0.05; and drug × time interaction, *F*_(1,36)_ = 10.239, *p* < 0.05; *post hoc* Bonferroni’s test, *ps* < 0.05; Fig. 1C). Consistent with the results of Fig. 1A, these observations suggest that ROL enhances the retrieval of IA memory and its maintenance. Importantly, the VEH and ROL groups displayed significantly increased crossover latency at test compared with re-exposure (paired *t*-test, *ps* < 0.05; Fig. 1C). Consistent with our previous studies [31–33], re-exposure to the light compartment enhanced IA memory in this task.

In contrast, systemic injection of NB001 impaired IA memory retrieval and its maintenance (two-way ANOVA: drug, *F*_(1,36)_ = 30.692, *p* < 0.05; time, *F*_(1,36)_ = 15.02, *p* < 0.05; and drug × time interaction, *F*_(1,36)_ = 17.531, *p* < 0.05; Fig. 1D). The NB001 group showed significantly shorter crossover latency at re-exposure and test than the VEH group (*post hoc* Bonferroni’s test, *ps* < 0.05), although the VEH group, but not the NB001 group, displayed significantly increased crossover latency at test compared with re-exposure (paired *t*-test, *p* < 0.05; Fig. 1D).

It is important to note that our previous studies have shown IA memory, but not contextual fear memory, is enhanced following memory retrieval through memory reconsolidation [19, 27, 29–33, 47–50]. Those differences in “reconsolidation effect” between IA memory and contextual fear memory are reflected by the results of two-way ANOVA (a significant effect of time and time × drug interaction was observed in the IA memory, but not contextual fear memory).

**Fig. 2:**
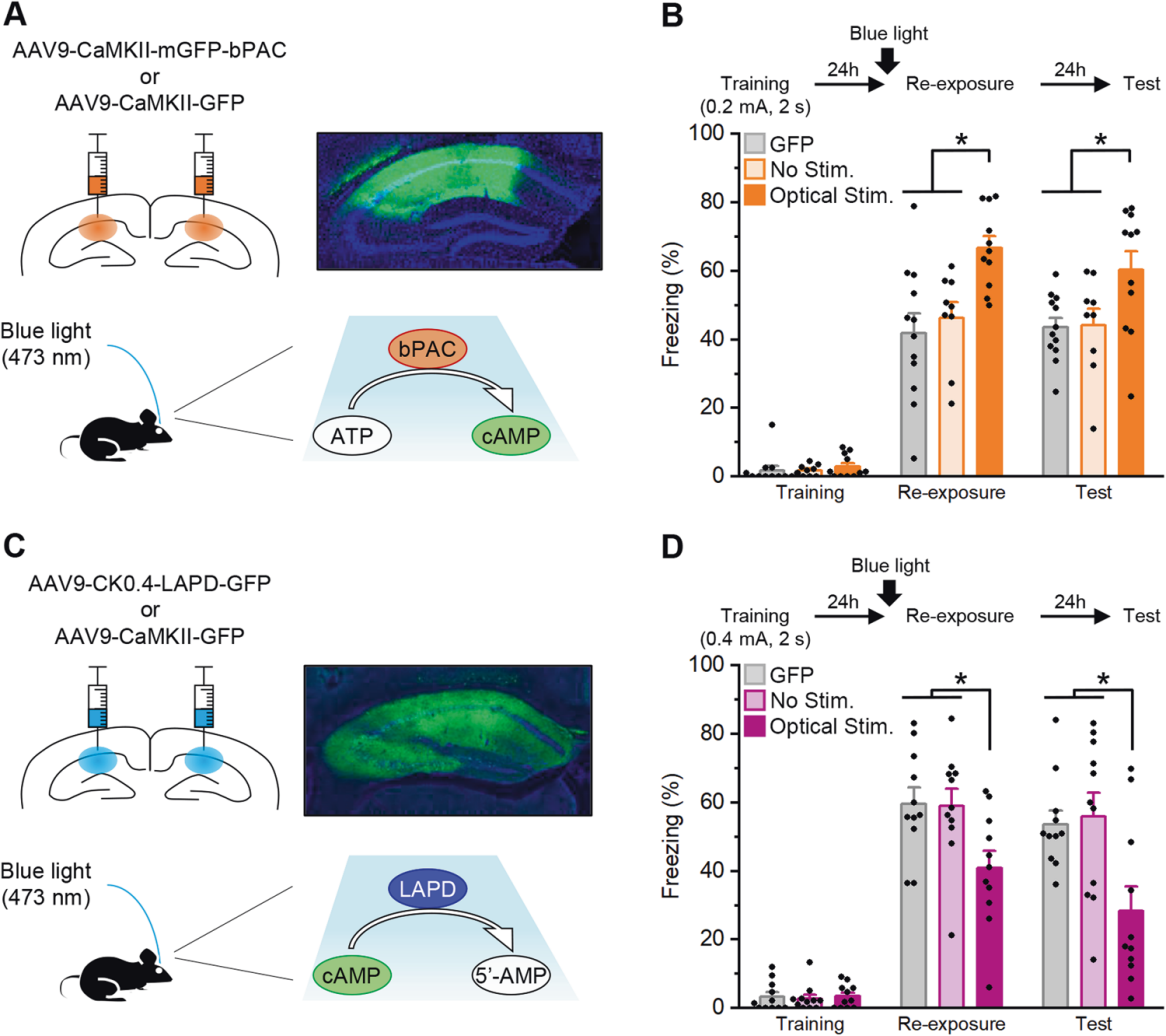
Effects of the optogenetic manipulation of cAMP levels in the dorsal hippocampus on fear memory retrieval and fate. **A**, **B** Effects of increased cAMP levels in the dorsal hippocampus by 473-nm blue light stimulation on contextual fear memory. **A** Schematic illustration of virus injection and mGFP expression in the dorsal hippocampus (upper images). Photostimulation of photoactivatable adenylyl cyclase (bPAC; lower image). **B** Effects of optogenetic increased cAMP levels in the dorsal hippocampus on contextual fear memory. GFP, *n* = 12; no stimulation (No Stim.), *n* = 9; optical stimulation (Optical Stim.), *n* = 11. **C**, **D** Effects of decreased cAMP levels in the dorsal hippocampus by 473-nm blue light stimulation on contextual fear memory. **C** Schematic illustration of virus injection and GFP expression in the dorsal hippocampus (upper images). Photostimulation of light-activated phosphodiesterase (LAPD; lower image). **D** Effects of optogenetic decreased cAMP levels in the dorsal hippocampus on contextual fear memory. GFP, *n* = 11; No Stim., *n* = 11; Optical Stim., *n* = 11. *p < 0.05; *post hoc* Bonferroni’s test. Error bars indicate SEM.

To investigate further the roles of cAMP levels in fear memory, we examined the effects of optogenetic manipulation of cAMP levels in the hippocampus. We used newly developed optogenetic probes that enable the generation or degradation of cAMP in a blue light-dependent manner. To examine the effects of increased cAMP levels, the mice received a micro-infusion of an AAV vector expressing bPAC under the control of the CaMKII promoter (AAV9-CaMKII-mGFP-bPAC or AAV9-CaMKII-GFP) into the dorsal hippocampus (Fig. 2A). We performed a similar experiment as in Fig. 1A, except that blue light was administered at 4 Hz (pulse width, 15 ms) using a 473-nm laser for 30 min from 40 min before re-exposure. The bPAC group froze significantly more in re-exposure and test compared to the GFP and No light control groups (two-way ANOVA: group, *F*_(2, 58)_ = 12.566, *p* < 0.05; time, *F*_(1, 60)_ = 0.362, *p* > 0.05; and group × time interaction, *F*_(2, 60)_ = 0.393, *p* > 0.05; *post hoc* Bonferroni’s test, *ps* < 0.05; Fig. 2B). These observations suggest that an optogenetic increase of cAMP levels in the hippocampus facilitates the retrieval and maintenance of contextual fear memory.

We then examined the effects of an optogenetic decrease of cAMP levels. The mice received a micro-infusion of AAV expressing LAPD under the control of the CaMKII promoter (AAV9-CK0.4-LAPD-GFP or AAV9-CaMKII-GFP) into the dorsal hippocampus (Fig. 2C). We performed a similar experiment as in Fig. 1B, except that blue light was administered at 4 Hz (pulse width, 15 ms) using a 473-nm laser for 30 min from 40 min before re-exposure. The LAPD group froze significantly less in re-exposure and test compared to the GFP and No light control groups (two-way ANOVA: group, *F*_(2,60)_ = 10.825, *p* < 0.05; time, *F*_(1,60)_ = 2.498, *p* > 0.05; and group × time interaction, *F*_(2,60)_ = 0.362, *p* > 0.05; *post hoc* Bonferroni’s test, *ps* < 0.05; Fig. 2D). These results suggest that an optogenetic decrease of cAMP levels in the hippocampus impairs the retrieval and maintenance of contextual fear memory.

It is important to note that the effects of optogenetic manipulations of bPAC and LAPD on cAMP-signal transduction in the hippocampus were examined. To do this, we measured the phosphorylation levels of CREB at serine 133 (pCREB levels) which is a target of protein kinase A (PKA) activated by cAMP. The mice expressing bPAC or LAPD showed significantly increased or decreased, respectively, pCREB levels in the CA1 region of the dorsal hippocampus 30 min after blue light stimulation for 30 min compared to the control group (Supplementary Fig. 1). These observations suggest that optogenetic manipulations of bPAC and LAPD are reflected by activation or inactivation, respectively, of cAMP-PKA signal transduction.

Collectively, these observations indicate that an increase or decrease of cAMP levels around retrieval enhances or impairs, respectively, the retrieval and subsequent maintenance of fear memory. Therefore, our observations suggest that the state of fear memory reflects cAMP levels.

### Comparison of human and mouse transcriptomes identifies a decrease in *PDE4B/Pde4b* mRNA levels in PTSD patients showing more severe reexperiencing symptoms and mouse hippocampus after retrieval

We then integratively analyzed transcriptome profiles associated with mouse fear memory retrieval and those associated with human reexperiencing symptoms.

**Fig. 3:**
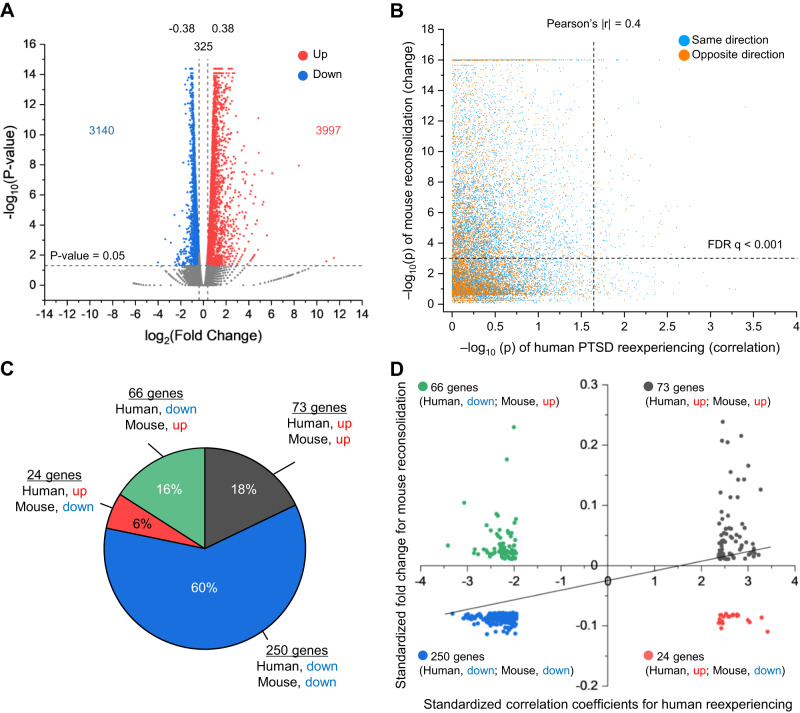
Mouse fear memory retrieval-associated transcriptome changes and human PTSD reexperiencing-associated transcriptome changes. **A** IA memory retrieval rapidly induced gene expression changes in the mouse dorsal hippocampus. Volcano plot showing 3997 upregulated (log_2_ fold change > 0.38, *p* < 0.05, red) and 3140 downregulated (log_2_ fold change < −0.38, *p* < 0.05, blue) genes in the dorsal hippocampus of the reactivated group compared to the non-reactivated group. **B** Relationship between human PTSD reexperiencing-associated expression changes and mouse fear memory retrieval-associated expression changes. All of the 16,721 genes analyzed in both humans (i.e., the female PTSD patients) and mice are included in this figure. The *x*-axis represents the -log_10_(*p*) of correlations between human PTSD reexperiencing symptoms and blood gene expression levels measured by microarray. The *y*-axis represents the -log_10_(*p*) of expression change after fear memory retrieval in mouse hippocampus measured by RNA-sequencing. Blue dots indicate those genes that show the same (consistent) direction of change in expression (i.e., either upregulation or downregulation) between humans and mice, while orange dots indicate those genes that show the opposite (inconsistent) direction of change in expression between humans and mice. **C**, **D** Consistency between human PTSD reexperiencing-associated expression changes and mouse fear memory retrieval-associated expression changes. **C** Pie chart showing the proportion of consistently vs. inconsistently regulated genes between human and mouse data for the 413 overlapping genes. The four (i.e., 2 × 2) patterns by upregulation and downregulation for humans and mice are distinguished. **D** Relationship between human PTSD reexperiencing-associated expression changes and mouse fear memory retrieval-associated expression changes for the 413 genes. The *x*-axis represents standardized correlation coefficients between human PTSD reexperiencing symptoms and blood gene expression levels. The *y*-axis represents standardized fold change after fear memory retrieval in mouse hippocampus.

For mouse transcriptome analysis, we performed similar behavioral experiments as in Fig. 1D and then RNA-seq analysis of the mouse dorsal hippocampus at 30 min following IA memory retrieval (re-exposure). We observed significantly increased (3997) and decreased (3140) mRNAs compared to the control group (non-reactivated; NR) that were trained, but not re-exposed to the light compartment during the re-exposure session (Fig. 3A). These observations suggest that IA memory retrieval rapidly induces gene expression changes in the dorsal hippocampus.

For human transcriptome analysis, the demographic and clinical characteristics of the PTSD patients and healthy controls are shown in Supplementary Table 1. All participants were female, on average aged in their mid-to-late 30 s and tertiary educated, and predominantly nonsmokers. Most patients developed PTSD after experiencing interpersonal violence. The patients had long duration of illness at the time of study participation; of the 32 patients, 6 had been ill for 6 months to 3 years, five had been ill for 3–5 years, and 21 had been ill for 5 years or more. The mean IES-R total score of 51.3 (standard deviation: 18.0) in patients suggested that their overall PTSD severity was moderate to severe, given that a score of 44 or more is used to indicate severe PTSD in many studies (reviewed in [51]). Most (78.1%) of the patients were receiving at least one class of psychotropic medication, including antidepressants (62.5%), anxiolytics (65.6%), antipsychotics (28.1%), mood stabilizers (12.5%), and hypnotics (46.9%).

The integrative analyses were first performed using 16,721 genes that were common to both transcriptome datasets, i.e., 48,908 human probes (genes) and 46,989 mouse genes. Genes that exceeded the hybrid thresholds of Pearson’s |*r*| > 0.40 for the human data and a false discovery rate (FDR) *q* < 0.001 for the mouse data consisted mostly of those showing a consistent direction of change in expression (Fig. 3B). The predominance of consistent over inconsistent direction of expression for those genes above the threshold indicates the enrichment of true (rather than chance) signals, which supported our integrative approach of analyzing human reexperiencing- and mouse fear memory retrieval-associated transcriptomic changes.

Consistency between the human and mouse transcriptomic changes was further investigated by scrutinizing genes that exceeded both thresholds. For the human data, 1377 probes with gene names were significantly correlated with reexperiencing symptom severity (Pearson’s *r*, *p* < 0.05). For the mouse data, 7462 genes exceeded the FDR threshold (*t*-test, *q* < 0.05). These analyses identified 413 overlapping genes (excluding one outlier that was >10 standard deviations above the mean value) that fulfilled both thresholds, most of which (i.e., 78%) displayed a consistent direction of expression between humans and mice (Fig. 3C). The correlation between “correlation coefficients of gene expression levels with reexperiencing symptoms in patients” and “fold change (FC) after retrieval in mice” for the 413 genes was highly significant (*r* = 0.49, *p* = 7.3E-26) (Fig. 3D).

**Fig. 4:**
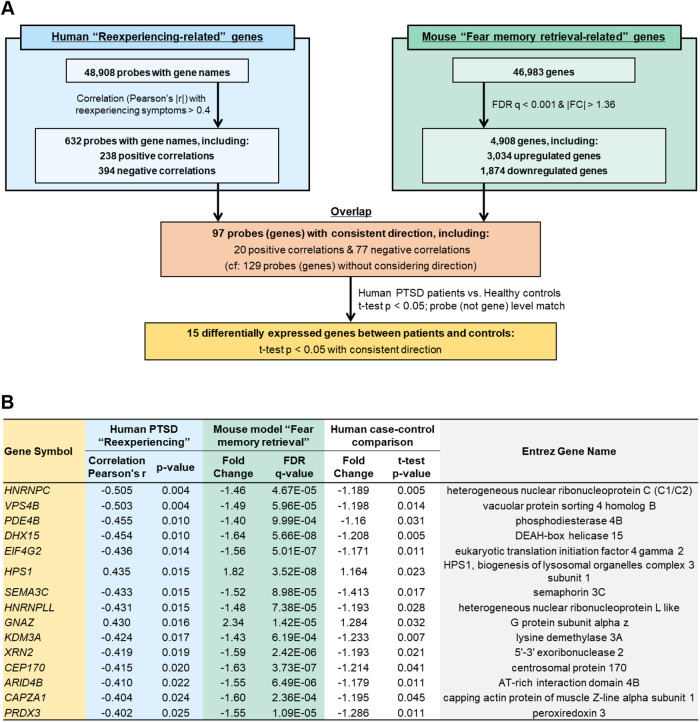
Integrative human and mouse transcriptome analysis. **A** Flow diagram of integrative human and mouse transcriptome analysis. The top left and right arms describe the flow of human and mouse transcriptome data analysis, respectively. The bottom two boxes indicate further selection steps by integrating the human and mouse data. The human data were examined at the probe level, that is, different microarray probes within the same gene were distinguished. **B** List of the 15 genes overlapping across all three datasets. In this figure, gene symbols are represented using human gene nomenclature, i.e., all letters are italicized and in upper-case (e.g., *PDE4B*).

We then performed the integrative transcriptome analysis to identify key genes; for the analytic flow, see Fig. 4A. For the human blood transcriptome data, the expression levels of 632 probes were significantly correlated with reexperiencing symptom severity (including 238 positive and 394 negative correlations) using the threshold of |*r*| > 0.40. For the mouse hippocampus transcriptome data, the expression levels of 4908 genes (3034 upregulated and 1874 downregulated) were significantly changed after fear memory retrieval to induce reconsolidation compared to no-retrieval control using the FDR threshold of *q* < 0.001 and |FC| > 1.36. These analyses identified 97 overlapping genes in both datasets with a consistent direction of expression (Supplementary Table 2). Of the 97 genes, 15 showed significant differences between PTSD patients and healthy controls with a consistent direction, using the *t*-test threshold of *p* < 0.05. The curated 15 genes overlapping across all three datasets included *PDE4B/Pde4b* (Fig. 4B).

**Fig. 5:**
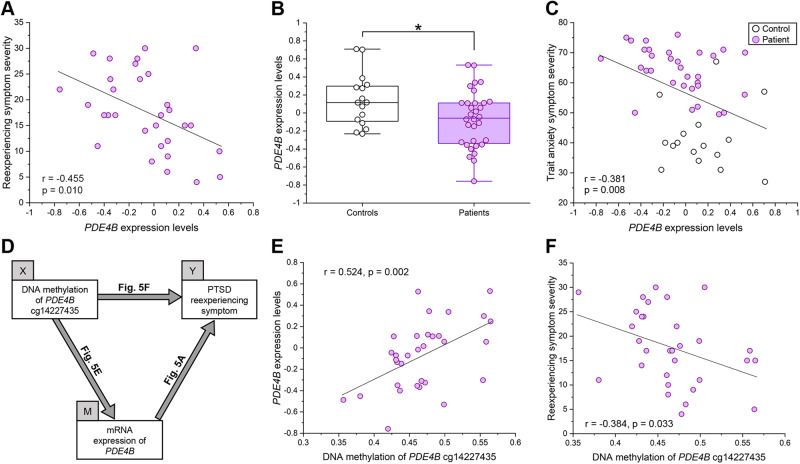
Relationship of *PDE4B* expression levels with PTSD diagnosis, symptoms, and DNA methylation levels in humans. **A**, **B**, **C** Relationship of *PDE4B* expression levels with PTSD diagnosis and associated symptoms in humans. Plots in purple indicate PTSD patients and those in white indicate healthy controls. **A** Scatterplot showing the correlation between *PDE4B* expression levels and reexperiencing symptoms in PTSD (*n* = 31). **B** Combined dot- and box-plot showing *PDE4B* expression levels in PTSD patients (*n* = 32) compared to healthy controls (*n* = 16). *p = 0.031. **C** Scatterplot showing the correlation between *PDE4B* expression levels measured by microarray and trait anxiety symptoms assessed with the State-Trait Anxiety Inventory-Trait scores in the total sample (*n* = 48). **D**, **E**, **F** Mediation model in which *PDE4B* expression levels mediate the relationship between *PDE4B* cg14227435 methylation status and PTSD reexperiencing symptoms in patients. **D** Schema of the mediation model tested. The independent variable (X) was cg14227435 methylation level. The dependent variable (Y) was reexperiencing symptom severity. The mediator variable (M) was *PDE4B* mRNA expression levels. **E** Scatterplot showing the correlation between cg14227435 methylation levels and *PDE4B* expression levels in patients (*n* = 32). **F** Scatterplot showing the correlation between cg14227435 methylation levels and reexperiencing symptom severity in patients (*n* = 31).

Specifically, *PDE4B/Pde4b* expression levels were significantly negatively correlated with PTSD reexperiencing symptoms (*r* = −0.46, *p* = 0.01; Spearman’s *ρ* = −0.49, *p* = 0.005), downregulated after fear memory retrieval in mice (FC = −1.4, *p* < 0.001), and lower in patients than in controls (*p* = 0.031) (Fig. 4B and Fig. 5A, B). The significant negative correlation of *PDE4B* expression levels with reexperiencing symptoms and the significantly lower expression levels in patients compared to controls were essentially unchanged after controlling for participants’ age in partial correlation analysis (*r* = −0.45, *p* = 0.01) and in analysis of covariance (*F*_(1,45)_ = 4.93, *p* = 0.031), respectively. In addition, *PDE4B* expression levels were significantly negatively correlated with trait anxiety (*r* = −0.38, *p* = 0.008) (Fig. 5C), but not with state anxiety (*p* = 0.09) or depressive symptoms (*p* = 0.11) in the total human sample.

Of the PDE gene family, *PDE4B* was expressed at remarkably high levels in human blood (Supplementary Fig. 2). Since cAMP signaling-associated genes have been implicated in PTSD [6, 7] and cAMP signaling facilitates fear memory retrieval, our observations suggest that reexperiencing symptoms in patients with PTSD may be associated with enhanced cAMP signaling via the downregulation of PDE4, which degrades cAMP.

**Fig. 6:**
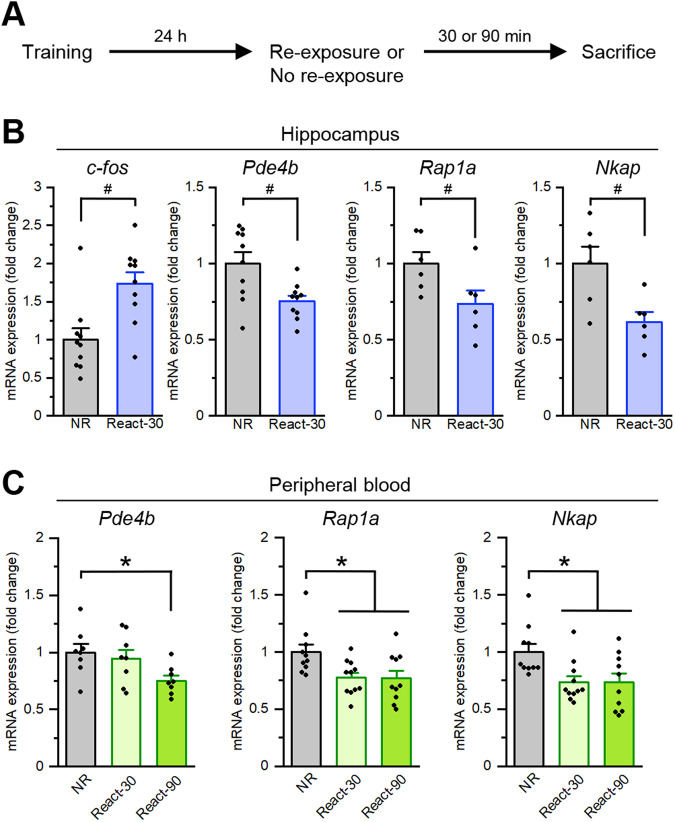
Changes of mRNA levels in the murine dorsal hippocampus and peripheral blood following IA memory retrieval. **A** Experimental design. **B** Changes of mRNA levels in the dorsal hippocampus. Non-reactivated (NR), *n* = 6–10; 30 min after reactivation (React-30), *n* = 6–10. **C** Changes of mRNA levels in peripheral blood. NR, *n* = 8–10; React-30, *n* = 8–11; 90 min after reactivation (React-90), *n* = 8–10. *p < 0.05, *post hoc* Bonferroni’s test; #*p* < 0.05, Student’s *t*-test. Error bars indicate SEM.

Correlation analysis of *PDE4B* expression levels within the human transcriptome data identified 519 probes for 483 unique genes that exceeded the threshold of Pearson’s |*r*| > 0.6 (Supplementary Table 3). Gene Ontology analysis using these 483 genes revealed that they were significantly overrepresented in several Gene Ontology terms, with the top signal being “activation of MAPKK activity” (Supplementary Table 4). Among the MAPK family genes, Ras-associated protein 1A (*RAP1A/Rap1a*) was strongly co-expressed with *PDE4B* (Supplementary Table 3) and significantly downregulated in relation to PTSD reexperiencing symptoms (Supplementary Table 2) and mouse fear memory retrieval (Fig. 6 and Supplementary Table 2).

### *PDE4B* DNA methylation status correlates with its mRNA expression levels and PTSD reexperiencing symptoms

We examined whether the DNA methylation patterns of *PDE4B* influenced the relationship between its mRNA expression levels and reexperiencing symptoms in the same human sample. For this purpose, we focused on all 125 CpG sites across *PDE4B* in a DNA methylation array and calculated correlations of their DNA methylation levels with reexperiencing symptom severity and *PDE4B* mRNA levels. Of the 125 CpG sites, five were significantly correlated with reexperiencing symptoms and mRNA expression (Supplementary Table 5). Moreover, the directions of these tripartite correlations among DNA methylation levels, mRNA levels, and reexperiencing symptoms were compatible for all five sites. These results suggest that *PDE4B* DNA methylation levels affect PTSD reexperiencing symptoms by altering its mRNA expression levels.

Of the five sites, we focused on cg14227435 (located in the gene body) that is assumed to be an enhancer by the ENCODE Consortium, and performed mediation analysis to further investigate the relationship between cg14227435 methylation status, *PDE4B* mRNA expression levels, and reexperiencing symptoms in patients (Fig. 5D). This mediation model was built based on the significant tripartite correlations between cg14227435 methylation levels, *PDE4B* mRNA expression levels, and reexperiencing symptoms (Fig. 5A, E, F). The results indicated complete mediation, with significant effects of methylation on expression (estimate: 3.86; standard error [SE] = 0.75, *p* < 0.001) and of expression on reexperiencing (estimate: −8.54; SE = 4.17, *p* = 0.040), and a nonsignificant effect of methylation on reexperiencing (estimate: −27.3; SE = 25.7, *p* = 0.29). The indirect effect was significant (estimate: −0.21; SE = 0.11, *p* = 0.049). These results support the mediation model in which *PDE4B* DNA methylation status affects PTSD reexperiencing symptoms by altering *PDE4B* mRNA expression levels (Fig. 5D).

### Decreased *Pde4b* mRNA levels in the murine hippocampus and blood after fear memory retrieval

We validated the mRNA expression changes in the mouse dorsal hippocampus following IA memory retrieval using qRT-PCR (Fig. 6). To do this, we performed similar experiments as in Fig. 1D and measured hippocampal mRNA levels at 30 min after reactivation [React-30 and NR groups] (Fig. 6A). We measured the mRNA levels of *Pde4b*, *Rap1a*, and *Nkap* since these genes showed marked downregulation in human blood and mouse hippocampus transcriptomes in relation to reexperiencing/fear memory retrieval. Similar to previous studies [31, 32, 52, 53], significantly increased *c-fos* mRNA levels were observed in the hippocampus of the React-30 group compared to the NR group (*t*-test, *p* < 0.05; Fig. 6B). Importantly, *Pde4b*, *Rap1a*, and *Nkap* mRNA levels were significantly decreased in the hippocampus of the React-30 group compared to the NR group (*t*-test, *ps* < 0.05; Fig. 6B). To examine the relationship of mRNA levels between the hippocampus and peripheral blood following IA memory retrieval in mice, we measured blood mRNA levels at 30 and 90 min after reactivation (Fig. 6C). Similarly with the results of the hippocampus, *Pde4b*, *Rap1a*, and *Nkap* mRNA levels were significantly decreased in the peripheral blood of the React-30 and/or -90 groups compared to the NR group (one-way ANOVA followed by *post hoc* Newman–Keuls test: *Pde4b*, F_(2,21)_ = 2.649, *p* < 0.05; *Rap1a*, F_(2,28)_ = 4.727, *p* < 0.05; *Nkap*, *F*_(2,28)_ = 3.674, *p* < 0.05; Fig. 6C), indicating that *Pde4b*, *Rap1a*, and *Nkap* mRNA expression levels in the hippocampus and peripheral blood were decreased following IA memory retrieval. Our results suggest that the IA memory retrieval-induced changes in these mRNA levels were consistent between the hippocampus and peripheral blood in mice.

## Discussion

The pathogenesis of PTSD includes genetic factors [54, 55]. Previous genetic studies have identified genes related to the cAMP signaling pathway as candidate PTSD-associated genes [6, 7]. These findings, together with our previous finding in mice that cAMP signaling pathway activation promotes memory retrieval [23], raise the possibility that cAMP levels may correlate with the symptoms of PTSD-associated with traumatic memory retrieval such as reexperiencing. To investigate this possibility, we examined the effects of the gain- and loss-of-function of the cAMP signaling pathway on hippocampus-dependent contextual fear and IA memories in mice. Pharmacological and optogenetic activation or inactivation of cAMP signaling facilitated or impaired, respectively, the retrieval and maintenance of these memories. These findings suggest that the state of the cAMP signaling pathway determines the state of fear memory. Importantly, our integrative transcriptome analysis identified the downregulated mRNA expression of PDE4B, an enzyme that degrades cAMP, in the peripheral blood of female PTSD patients with reexperiencing symptoms and in the hippocampus of mice following fear memory retrieval. Furthermore, *PDE4B* mRNA expression was downregulated in the hippocampus and peripheral blood of mice following fear memory retrieval. Since the downregulation of *PDE4B* mRNA expression leads to the activation of the cAMP signaling pathway via a decrease in cAMP degradation, our findings raise the possibility that the downregulation of *PDE4B* expression may be associated with the mechanisms for reexperiencing symptoms in PTSD patients through the activation of the cAMP signaling pathway.

cAMP signaling pathway plays critical and positive roles in learning and memory and neural plasticity from invertebrate to mammalian animal models [56–64]. Recently our study showed that this signaling pathway activation promotes memory retrieval via the activation of protein kinase A (PKA) and the phosphorylation of AMPA glutamate receptor GluA1 at serine 845 by PKA [23]. Our mouse behavioral studies showed that the gain- or loss-of-function of the cAMP signaling pathway enhanced or blocked, respectively, fear memory retrieval. These findings support the hypothesis that the state of the cAMP signaling pathway determines fear memory retrieval.

Memory reconsolidation is thought to be a process of updating and/or strengthening consolidated memories [13–16]. Our previous studies have shown that memory retrieval strengthens IA memory via memory reconsolidation [31–33]. On the other hand, the cAMP signaling pathway activates the CREB-mediated transcription required for memory consolidation and reconsolidation through the phosphorylation of CREB [19]. Importantly, the upregulation of CREB-mediated transcription improves memory consolidation [28]. Moreover, the activation of PKA signaling enhances the reconsolidation of amygdala-dependent cued fear memory [22]. In the present study, memory maintenance was facilitated or impaired by activated and inactivated cAMP signals, respectively, before memory retrieval. Additionally, optogenetic manipulations of bPAC and LAPD increased or decreased respectively, pCREB levels in the dorsal hippocampus (Supplementary Fig. 1). Therefore, our observations suggest that activation or inactivation of cAMP signaling enhances or impairs, respectively, the reconsolidation of fear memory through CREB-mediated transcription, thereby modulating memory maintenance.

We observed that IA memory retrieval decreased *Pde4b* mRNA levels in mouse hippocampus and peripheral blood, suggesting synchronous gene expression regulation between brain and blood cells, although the molecular mechanism of this memory retrieval-induced regulation of expression needs to be clarified. Importantly, the reexperiencing symptoms of PTSD patients were correlated with decreased *PDE4B* mRNA levels in peripheral blood. Therefore, our observations suggest that PTSD patients exhibiting reexperiencing symptoms may have reduced *PDE4B* expression in the brain. More importantly, since cAMP levels are increased by decreased PDE4B levels, it is possible that the downregulation of PDE4B contributes to the enhancement of fear memory retrieval via an increase of cAMP levels. Therefore, elevated cAMP levels mediated by the decreased expression of PDE4B may have a significant impact on the pathophysiology of PTSD, especially reexperiencing symptoms. In the future, PDE4B expression levels in the brain of PTSD patients with pronounced reexperiencing symptoms should be examined.

The cAMP signaling pathway is regulated by abundant signal transduction molecules. As discussed above, our findings suggest the possible underlying mechanism of PTSD by the activation of this pathway. Importantly, previous studies have examined PTSD-associated genes related to the cAMP signaling pathway, such as *ADCYAP1*, *ADCYAP1R1*, and *CRHR1* [6, 7]. Moreover, a recent large-scale genome-wide association study of PTSD patients also listed *PDE4B* as a significant locus [7]. These findings indicate that the development of PTSD may be influenced by genes related to the cAMP signaling pathway; therefore, the molecules involved in this pathway could be candidate causative molecules of PTSD. PTSD may not be associated with mutations in only specific genes, but rather with mutations in a group of genes enabling the activation of the cAMP signaling pathway. It is important to clarify the etiology of PTSD in terms of mutations of cAMP signaling-related genes.

The pathogenesis of PTSD is thought to involve multiple genetic factors, environmental components, and their complex interactions [54, 55, 65, 66]. Since transcriptome profiles reflect the dynamic nature of genomic responses to environmental stimuli, gene expression analysis can be a useful approach for understanding the etiology of PTSD. A number of transcriptome studies have been conducted on individuals with PTSD, but their results are not uniform [67–69]. Such inconsistency may be due, at least in part, to the well-known phenotypic heterogeneity of PTSD, which may be underlined by heterogeneous molecular mechanisms [70]. In the present study, we focused on reexperiencing symptoms as a single and core symptomatology, rather than the diagnosis itself. Since the mechanisms for fear memory regulation are thought to be similar between humans and other animals [17–21], we investigated the transcriptome profiles of PTSD patients by integrating human reexperiencing- and mouse fear memory retrieval-associated transcriptome profiles. The concordance between human and mouse transcriptome changes indicates the validity of our integrative human-mouse transcriptome data analysis, which is similar to previous findings [71]. Specifically, the expression of *PDE4B*/*Pde4b* was downregulated in relation to reexperiencing symptoms in human PTSD patients and fear memory retrieval in mice. Furthermore, synchronous downregulation of *Pde4b* mRNA expression was observed between the brain and peripheral blood cells following fear memory retrieval in mice. These findings suggest that *PDE4B*/*Pde4b* mRNA expression is downregulated in a traumatic memory retrieval/reexperiencing-dependent fashion similarly in mice and humans. Molecular mechanism(s) underlying such synchrony in gene expression between the brain and the blood is not fully elucidated, but there are some studies that have investigated this issue. Specifically, a number of studies have compared blood and brain gene expression profiles in humans and in animals, which overall demonstrate a considerable extent of correlations between expression patterns in these two tissues (reviewed in [72]). Notably, concordant expression of core genes between blood and brain has been shown in rodent models of PTSD [73, 74]. Additionally, correlations between expression patterns in peripheral blood and hippocampus in mouse models of major depressive disorder and Alzheimer’s disease were observed [75, 76]. It is also shown that genetic effects on gene expression are highly correlated between brain and blood samples, especially with respect to the top-associated *cis*-expression quantitative trait loci [77]. This suggests that the consistent gene expression in the brain and the blood may partly be caused by the common genetic effects on gene expression across these two tissues.

We also examined the genes co-expressed with *PDE4B* using human blood transcriptome data. Gene Ontology analysis of the co-expressed genes identified ontology related to MAPK activation as the top signal, and among the MAPK family, *RAP1A* was strongly co-expressed with *PDE4B*. This finding accords with evidence suggesting crosstalk between cAMP and MAPK signaling [78]. cAMP exerts its action largely via the ubiquitously expressed cAMP-dependent PKA. Besides the classic PKA, the exchange proteins directly activated by cAMP (EPACs), also known as cAMP-regulated guanine nucleotide exchange factors, function as key effectors of cAMP [79]. Importantly, RAP1 is activated by EPACs [80, 81]. While EPACs are involved in multiple physiological processes, they play a major role in central nervous system functions including learning and memory [79]. Notably, EPACs induce dynamic remodeling and depression of synapses [82] and play a role in hippocampus-dependent contextual memory retrieval together with cAMP-PKA signaling [24]. Similarly, Epac 2 knockout mice showed an impairment of contextual fear memory [83]. In line with these findings, our data show that *RAP1A*/*Rap1a* expression was consistently downregulated in human PTSD reexperiencing and mouse fear memory retrieval. It is possible that *RAP1A* is involved in the mechanism by which cAMP-signal transduction is associated with reexperiencing symptoms.

Our results further suggest that the DNA methylation patterns of *PDE4B*, particularly the methylation status of cg14227435, regulate its mRNA expression levels to affect reexperiencing symptoms. According to the ENCODE SCREEN database [84], cg14227435 is a candidate *cis*-regulatory element that is assumed to act as a distal enhancer. Notably, cg14227435 showed a significant *positive* correlation with *PDE4B* expression. This result accords with the finding that DNA methylation in enhancer regions can promote gene expression [85]. In line with our results, a previous study using placental tissue reported that the DNA methylation levels at a CpG site located in the *PDE4B* gene body were significantly positively correlated with *PDE4B* mRNA expression [86].

There were several limitations to the current study. Firstly, the sample size of the human transcriptome study was not very large. Secondly, since we included only female participants in the human study it remains unclear whether the present findings might be specific to female PTSD patients or common to both sexes, given the evidence for sex differences in the etiology of PTSD [87]. Thirdly, the effects of pharmacological and optogenetic up-or-downregulation of cAMP signaling on memories other than fear memory have not been evaluated in mouse experiments.

In conclusion, this study shows that increased cAMP levels promote memory retrieval and that *PDE4B* expression is downregulated in the hippocampus and peripheral blood in mice after fear memory retrieval and peripheral blood of PTSD patients showing more severe reexperiencing symptoms. These findings suggest that the increased cAMP levels caused by the downregulation of *PDE4B* mRNA expression enhance traumatic memory, thereby playing a key role in the reexperiencing symptoms of PTSD patients as a functional index of these symptoms.

## Supplementary information

Supplementary Information

Supplementary Tables S1–S6

## Data Availability

The data that support the findings of the current manuscript are available from the corresponding authors, HH and SK, upon reasonable request. Human microarray data have been deposited to the Gene Expression Omnibus (GEO) database repository with the dataset identifier GSE199841. Mouse RNA-sequencing data have been deposited to the DDBJ Sequence Read Archive (DRA) and are available at the accession number DRA013665.
