# Supplementary material for: Fear memory regulation by the cAMP signaling pathway as an index of reexperiencing symptoms in posttraumatic stress disorder
Source: Mol Psychiatry. 2024 Feb 27;29(7):2105–16. doi: 10.1038/s41380-024-02453-4 (PMC11408251; doi:10.1038/s41380-024-02453-4)
Supplement: Supplementary file 1 — Supplementary Information [file 41380_2024_2453_MOESM1_ESM.docx]

**Supplementary Information**

**Fear memory regulation by the cAMP signaling pathway as an index of reexperiencing symptoms in posttraumatic stress disorder**

Hiroaki Hori*#, Hotaka Fukushima*, Taikai Nagayoshi*, Rie Ishikawa*, Min Zhuo, Fuyuko Yoshida, Hiroshi Kunugi, Kenichi Okamoto, Yoshiharu Kim#, Satoshi Kida#

*co-first

# corresponding

**Supplementary Methods**

**Mouse study**

***Mice***

All animal experiments were conducted according to the *Guide for the Care* and *Use of Laboratory Animals* (Japan Neuroscience Society and Tokyo University of Agriculture) and were approved by the Animal Care and Use Committee of Tokyo University of Agriculture (authorization #2021031). All surgical procedures were performed under Nembutal anesthesia and every effort was made to minimize suffering. Male C57BL/6N mice were obtained from Charles River (Yokohama, Japan). The mice were housed in cages of 5 or 6, maintained on a 12-h light/dark cycle, and allowed access to food and water *ad libitum*. The mice were at least 8 weeks of age when tested. Testing was performed during the light phase of the cycle. All experiments were conducted blind to the treatment condition of the mice (n = 225 in total).

***Drugs***

The phosphodiesterase 4 (PDE4) inhibitor rolipram (ROL; Tocris Bioscience, UK) [1] was dissolved in dimethyl sulfoxide (DMSO; Wako, Osaka, Japan) and then diluted with distilled water for systemic injection experiments (Fig. 1A, C). Mice were systemically injected with ROL (0.1 mg/kg b.w.) or vehicle (VEH) 30 min before re-exposure. The adenylyl cyclase 1 (AC1) inhibitor NB001 [2] was dissolved in saline for systemic injection experiments (Fig. 1B, D). Mice were systemically injected with NB001 (30 mg/kg b.w.) or VEH twice every hour from 2 h before re-exposure.

***Contextual fear conditioning task***

The mice were trained and tested in conditioning chambers (17.5 × 17.5 × 15 cm) (O’Hara & Co., Ltd., Tokyo, Japan) that had a stainless-steel grid floor through which a footshock could be delivered [3-7]. Training consisted of placing the mice in the chamber and delivering a single electric footshock [2 s duration; 0.2 mA (Fig. 1A and Fig. 2B) or 0.4 mA (Fig. 1B and Fig. 2D)], at 148 s later, and the mice were returned to their home cage at 30 s after the footshock (training).

To examine the effects of increased or decreased cAMP levels on retrieval and maintenance of contextual fear memory (Fig. 1A, B and Fig. 2), the mice were trained as described above, and at 24 h later, the mice were placed back in the conditioning chamber for 3 min (re-exposure). At 24 h after re-exposure, the mice were once again placed back in the conditioning chamber for 5 min and freezing was assessed (test). Memory was assessed as the percentage of time spent freezing in the training context. Freezing behavior (defined as a complete lack of movement, except for respiration) was measured automatically by video (O’Hara & Co., Ltd., Tokyo, Japan) as described previously [8].

***Inhibitory avoidance (IA) task***

The step-through IA apparatus (O’Hara & Co., Ltd., Tokyo, Japan) consisted of a box with separate light and dark compartments (both 15.5 × 12.5 × 11.5 cm). The light compartment was illuminated by a fluorescent light (2500 lux) [9-12]. During the training sessions, each mouse was allowed to habituate to the light compartment for 30 s, and the guillotine door was raised to allow access to the dark compartment. Latency to enter the dark compartment was considered as a measure of acquisition. As soon as the mouse had entered the dark compartment, the guillotine door was closed. After 5 s, a footshock [0.1 mA (Fig. 1C) or 0.2 mA (Fig. 1D)] was delivered for 2 s (training).

To examine the effects of increased or decreased cAMP levels on retrieval and maintenance of IA memory (Fig. 1C, D), the mice were trained as described above, and at 24 h later, the mouse was placed back in the light compartment until it entered the dark compartment without a footshock (re-exposure). Memory was assessed twice at 48 h after the re-exposure as the crossover latency for the mouse to enter the dark compartment when replaced in the light compartment, as in reactivation (test).

***Viruses***

A photoactivatable adenylyl cyclase (bPAC) was constructed as previously reported with S27A mutation by PCR [13-15]. A light-activated phosphodiesterase (LAPD) [16] with a catalytic domain of *Homo sapiens* phosphodiesterase 4 (NM_002600) was synthesized (GenScript, New Jersey, USA). AAV9-CaMKII-mGFP-bPAC (titer: 1.21 × 10^13^ VG/mL), AAV9-CK0.4-LAPD-GFP (titer: 3.16 × 10^13^ VG/mL), AAV9-CaMKII-GFP (titer: 2.98 × 10^13^ VG/mL) were packaged and purified by SignaGen laboratories (SL100863 and SL100864, Rockville, MD, USA).

***Virus injection and optical fiber implantation***

For virus injections, the stereotaxic injection of adeno-associated virus (AAV) vectors was performed in a biological safety cabinet. Mice were anesthetized with a combination of medetomidine-midazolam-butorphanol anesthesia and placed in a stereotaxic frame. The skull was exposed and a small portion of the skull over dorsal hippocampus was removed bilaterally with a drill. AAVs (0.3 μL/site at a speed of 0.1 μL/min) were injected into the mouse brains using glass capillary pipettes [The anteroposterior (A/P) -1.6 mm, mediolateral (M/L) ±1.6 mm, dorsoventral (D/V) (from Dura) -1.6 mm] pulled with a micropipette puller (P-87, Sutter Instruments, Novato, CA, USA). After the injection, the glass pipettes were left in place for another 5 min before being slowly lifted up and removed. The mice were sutured, and antibiotic ointment was applied. Mice were then kept on a warm heater for recovery. At 1 week after the virus injections, a stainless-steel guide cannula (22 gauge) was implanted into the dorsal hippocampus (-1.6 mm, ±1.6 mm, -1.6 mm), under a combination of medetomidine-midazolam-butorphanol anesthesia, using standard stereotaxic procedures. Surgery was performed as described previously [4-7,9-12]. The mice were allowed to recover for 1 week after surgery and then subjected to behavioral analyses. Stereotaxic coordinates for the dorsal hippocampus placement were based on the brain atlas of Franklin and Paxinos [17].

***Optogenetic manipulation of cAMP levels in the dorsal hippocampus***

To examine the effects of increased or decreased cAMP levels on fear memory, mice received a micro-infusion of an AAV vector expressing a photoactivatable adenylyl cyclase (bPAC) (AAV9-CaMKII-mGFP-bPAC or AAV9-CaMKII-GFP; Fig. 2A, B) or a light-activated phosphodiesterase (LAPD) (AAV9-CK0.4-LAPD-GFP or AAV9-CaMKII-GFP; Fig. 2C, D) under the control of the CaMKII promoter into the dorsal hippocampus, respectively [15]. The blue light was stimulated at 4 Hz (pulse width, 15 ms, ~1.0 mW, Lucir Inc., Tsukuba, Japan) using a 473-nm laser for 30 min from 40 min before the re-exposure.

Successful transduction of the hippocampus region was confirmed histologically by native GFP fluorescence. Only mice showing bilateral GFP expression in the hippocampus were included in subsequent data analyses.

***Measurement of phosphorylated CREB levels***

To examine the effects of increased or decreased cAMP levels on CREB phosphorylation, mice received a micro-infusion of an AAV vector expressing bPAC, LAPD or GFP under the control of the CaMKII promoter into the dorsal hippocampus, respectively, and then a stainless-steel guide cannula was implanted into the dorsal hippocampus as described above. The blue light stimulation was performed for 30 min as described above, and at 30 min later, mice were perfused and used for immunohistochemical staining of phosphorylated CREB positive cells.

***Immunohistochemistry***

Immunohistochemistry was performed as described previously [11,18]. After anesthetization, all mice were perfused with 4% paraformaldehyde. The brains were removed, fixed overnight, transferred to 30% sucrose, and stored at 4℃. Coronal sections (30 µm) were cut in a cryostat. Free-floating sections were treated with 1%H_2_O_2_ and incubated overnight with a rabbit polyclonal anti-phospho-CREB (serine 133; S133) antibody (1:2000; #06-519, Millipore) in blocking solution (phosphate-buffered saline plus 1% goat serum albumin, 1 mg/ml bovine serum albumin). The sections were washed with phosphate-buffered saline and incubated with horseradish peroxidase-conjugated donkey anti-rabbit IgG (1:500; #711-036-152, Jackson ImmunoResearch) for 1 h at room temperature. pCREB signals were amplified by biotin tyramide and visualized using Alexa Fluor 568-conjugated streptavidin (#S11226, Invitrogen). The sections were mounted on slides and coverslipped using mounting medium with DAPI (VECTASHIELD).

***Quantification*** ***of pCREB^+^ cells***

Quantification was performed as described previously [11,18]. Structures were defined anatomically according to the atlas of Franklin and Paxinos [17]. All immunoreactive neurons were counted by an experimenter blind to the treatment condition. Fluorescence images were acquired using a confocal microscope (TCS SP8; Leica, Wetzlar, Germany). Confocal 2-μm z-stack images were obtained using LAS AF software (Leica). Equal cutoff thresholds were applied to all slices. We quantified the number of pCREB^+^ and GFP^+^ cells using a 40× objective. For quantification of pCREB^+^ cells in the field of view within the hippocampus (290×290 mm; bregma between -1.46 and -1.82 mm) across at least two sections, computerized image analyses were performed using WinROOF version 5.6 software (Mitani Corporation, Fukui, Japan).

***RNA analysis***

Mouse hippocampal RNA analyses were performed as described previously [6,19]. Mice were sacrificed by cervical dislocation. The dorsal hippocampus (bregma between -1.46 and -2.18 mm) was dissected by Rodent Brain Matrices (MUROMACHI KIKAI Co., Ltd, Tokyo, Japan) and snap‐frozen in liquid nitrogen. Total RNA was prepared from mouse dorsal hippocampus region using the RNeasy Mini Kit (Qiagen, Valencia, CA, USA). To analyze the peripheral blood mRNA, the mice were anesthetized before blood collection by cardiac puncture [20]. Total RNA from peripheral blood was isolated using the RNAprotect Animal Blood Tubes (Qiagen) and RNeasy Protect Animal Blood Kit (Qiagen) according to the manufacturer’s instructions.

Quantitative reverse transcription PCR (qRT-PCR) was performed as described previously [6,19,21]. Total RNA (500 ng) was reverse transcribed using Superscript Ⅲ reverse transcriptase (Invitrogen) and an oligo dT primer. qRT-PCR was performed with the ABI PRISM 7000 (Applied Biosystems, CA, USA) using SYBR Green PCR Master Mix (Thermo Fisher Scientific) according to the manufacturer's protocol. The reaction was first incubated at 50 °C for 2 min, then at 95 °C for 10 min, followed by 40 cycles of 95 °C for 15 s and 60 °C for 1 min. Amplification of a single PCR product was confirmed by monitoring the dissociation curve. Amplification curves were visually inspected to set a suitable baseline range and threshold level. The relative quantification method was employed for the quantification of target molecules according to the manufacturer’s protocol, where the ratio between the amount of each target molecule and a reference molecule within the same sample was calculated. All measurements were performed in triplicate. The levels of *Gapdh* mRNA were used to normalize the relative expression levels of target mRNA. The primer sequences for qRT-PCR analyses are listed in Supplementary Table 6.

***RNA-sequencing***

RNA-sequencing was performed as described previously [6,19]. Total RNA from mouse dorsal hippocampus was isolated using the RNeasy Mini Kit (Qiagen). They were subjected to RNA-seq analysis for each experimental group (n = 5 animals per pool). After RNA quality check [all RNA integrity number (RIN) values > 8.0] with the RNA nano kit (Agilent Technologies) on Agilent Bioanalyzer (Agilent Technologies), 1 μg of total RNA was used for preparing cDNA libraries with the TruSeq RNA Sample Preparation Kit v2 (Illumina, San Diego, CA, USA). The derived cDNA libraries were analyzed on an Agilent Bioanalyzer with DNA 1000 Kit and quantified by qPCR using the KAPA Library Quantification Kit (KAPA Bio systems, Wilmington, MA, USA). cDNA libraries were pooled in lanes and clusters were generated on a cBot (Illumina) to obtain 100‐bp single reads in a HiSeq 2500 sequencer (Illumina). Demultiplexed fastq files were generated using bcl2fastq ver. 2.18 (Illumina). Filtering, mapping, and differential expression analysis were performed using the CLC Genomics Workbench software ver. 9.5 (Qiagen). The raw sequence reads were filtered to exclude adapter sequences, ambiguous nucleotides, and low-quality sequences and the retained sequences were aligned against the mouse genome (mm10). RNA sequencing data have been deposited to the DDBJ Sequence Read Archive (DRA) and are available at the accession number DRA013665.

***Statistical analysis***

One-way analysis of variance (ANOVA) followed by *post hoc* Newman-Keuls test and 2-way ANOVA followed by *post hoc* Bonferroni’s comparisons were used to analyze the effects of drug, time, and group. A paired *t*-test was used to analyze the differences in crossover latency within each group between two sessions (re-exposure vs. test). A Student’s *t*-test was used to analyze differences in mRNA expression levels.

**Human study**

***Participants***

The human study was conducted at three institutes: National Center of Neurology and Psychiatry, Tokyo Women’s Medical University, and Nagoya City University. This study was approved by the ethics committee of each institute involved, and was conducted in accordance with the Declaration of Helsinki. After description of the study, written informed consent was obtained from all participants.

The sample was identical to the one employed in our previous study [22]. All patients had been diagnosed as having PTSD by their attending clinicians. The experience of traumatic events and diagnosis of PTSD were confirmed by the Posttraumatic Diagnostic Scale (PDS) [23], a well-established self-administered scale. For PTSD diagnosis, the PDS shows very high concordance rate (i.e., 95.1%, κ = 0.90) [24] with the Clinician-Administered PTSD Scale [25], a gold-standard structured interview. The Mini International Neuropsychiatric Interview (MINI) [26] was also administered to identify any other Axis-I disorders as well as PTSD.

***Symptom assessment***

PTSD symptoms of the patients were assessed using the validated Japanese version [27] of the Impact of Event Scale-Revised (IES-R) [28], a 22-item self-report questionnaire measuring the three core PTSD symptom clusters: reexperiencing (intrusion), avoidance, and hyperarousal. Each item is scored on a 5-point scale of symptom intensity, with higher scores indicating greater PTSD symptoms. There was one patient who did not complete this questionnaire, and valid IES-R data were obtained from 31 patients.

Anxiety symptoms were assessed using the validated Japanese version [29] of the State-Trait Anxiety Inventory (STAI) [30], a self-report questionnaire widely used to assess anxiety. It consists of two subscales for trait (STAI-T) and state (STAI-S) anxiety, both of which comprise 20 items that are scored on a 4-point scale from 1 to 4; higher scores indicate greater anxiety.

Depression symptoms were assessed using the validated Japanese version [31] of the Beck Depression Inventory-II (BDI-II) [32], a 21-item self-report questionnaire widely used to measure depression severity during the past two weeks. Each item is scored on a 4-point scale from 0 to 3, with higher scores indicating more severe depressive symptoms.

***Blood sampling***

Collected blood was used for RNA analyses and also for DNA methylation analysis. For the RNA analyses, venous blood collected in PAXgene Blood RNA Tubes (PreAnalytiX) from each participant was incubated at room temperature for 24 h for RNA stabilization, and then stored at –80°C. RNA was extracted from whole blood according to the manufacturer’s guidelines, using the PAXgene Blood RNA System Kit (PreAnalytiX, Hombrechtikon, Switzerland). For the DNA analysis, venous blood was collected in EDTA tubes and DNA was extracted from buffy coat using Maxwell 16 Blood DNA Purification Kit.

***Microarray experiment and transcriptome analysis***

This study utilized the microarray-derived transcriptome data used in our previous study, in which only hypothesis-driven, proinflammatory-status stratified gene expression profiling was conducted [22]; however, the present study is the first time that the data are analyzed in a hypothesis-free, data-driven manner. Details of the microarray experiments are described in our previous paper [22].

Briefly, quality and quantity of total RNA samples were determined using a NanoDrop ND-1000 spectrophotometer (NanoDrop Technologies, Inc., Wilmington, DE, USA) and an Agilent 2100 Bioanalyzer (Agilent Technologies, Tokyo, Japan). All the 48 RNA samples showed optical density 260/280 ratios of 1.6 or more and RIN of 6.3 or more. Cy3-labelled cRNA was synthesized from 100 ng of total RNA using an Agilent Low Input Quick Amp Labeling Kit (Agilent Technologies). The microarray experiment for 48 samples, including hybridization and scanning, was performed on the same day under the same conditions.

Transcriptome measurements were performed using Agilent SurePrint G3 v3 human GE 8×60K microarrays (Agilent Technologies) that contain 58,341 probes, including more than 26,000 mRNA probes and 30,000 non-coding RNA (mostly long non-coding RNA) probes. Raw signal data were analyzed using GeneSpring GX software, version 14.8 (Agilent Technologies). The raw signal values were thresholded to 1.0, and log base 2-transformation was performed. The 75th percentile shift normalization and baseline transformation with the median of all samples were then conducted as recommended in the GeneSpring manual. The normalized data were used for all statistical analyses. Microarray data have been deposited to the Gene Expression Omnibus (GEO) database repository with the dataset identifier GSE199841.

***DNA methylation analysis***

Quality and quantity of total DNA samples were determined using NanoDrop 2000c Spectrophotometers (Thermo Fisher Scientific) and Qubit 2.0 Fluorometer (Thermo Fisher Scientific). Quality of the extracted genomic DNA was confirmed by agarose gel electrophoresis. All the 48 samples had OD260/280 ratios ≥ 1.8 and OD260/230 ratios ≥ 1.8. 500ng of genomic DNA was bisulfite converted using EZ DNA Methylation Kit (Zymo Research).

DNA methylation levels were measured with Infinium MethylationEPIC BeadChip that covers over 850,000 methylation sites, according to Infinium HD Methylation Protocol Guide, Manual Protocol (15019519 v01) (Illumina). Hybridization and processing were performed according to manufacturer’s instructions. The BeadChip was scanned on the Illumina iScan system, and background subtraction and normalization to internal controls were then performed with the GenomeStudio software Methylation Module (Illumina). The output of the GenomeStudio analysis is a beta-value for each CpG site interrogated.

***Data analysis***

When comparing transcriptome data between humans and mice, gene symbols were used to match the same genes between the two species; for example, human “*PDE4B*” and mouse “*Pde4b*” were considered as the same gene.

Coexpression analysis of a target gene was performed using all the microarray probes in the human total sample (i.e., patients and controls combined: n = 48). Specifically, correlations of expression levels between a target gene probe and the other 58,340 probes were calculated using Pearson’s r, and those genes that were highly coexpressed (i.e., |r| > 0.6) with the target gene were identified. Next, significantly overrepresented Gene Ontology (GO) terms for the identified coexpressed genes were examined using the Database for Annotation, Visualization and Integrated Discovery (DAVID) v6.8 functional enrichment tool [33]. The GO terms were analyzed by GOTERM BP_DIRECT on the DAVID functional annotation tool, and those terms with a Benjamini-Hochberg corrected p value of < 0.05 were considered significant.

Mediation analysis was used to explore the potential mediation of *PDE4B* mRNA expression levels that might underlie the relationship between DNA methylation levels of *PDE4B* and PTSD reexperiencing symptoms. The independent variable (X) in this mediation model was the methylation level, given the temporal precedence and putative causality. The dependent variable (Y) was reexperiencing symptom severity. The mediator variable (M) was mRNA expression level. This analysis was conducted with the Mplus version 7 [34], using the following command:

VARIABLE: NAMES = X Y M; USEVARIABLES = X Y M;

ANALYSIS: TYPE = GENERAL; ESTIMATOR = ML; BOOTSTRAP = 2000;

MODEL: Y ON M X; M ON X;

MODEL INDIRECT: Y IND M X;

OUTPUT: STANDARDIZED(STDYX) CINTERVAL(BCBOOT);

**References**

1 Rutten K, Prickaerts J, Blokland A. Rolipram reverses scopolamine-induced and time-dependent memory deficits in object recognition by different mechanisms of action. Neurobiol Learn Mem. 2006;85(2):132-8.

2 Wang H, Xu H, Wu LJ, Kim SS, Chen T, Koga K, et al. Identification of an adenylyl cyclase inhibitor for treating neuropathic and inflammatory pain. Sci Transl Med. 2011;3(65):65ra3.

3 Suzuki A, Josselyn SA, Frankland PW, Masushige S, Silva AJ, Kida S. Memory reconsolidation and extinction have distinct temporal and biochemical signatures. J Neurosci. 2004;24(20):4787-95.

4 Suzuki A, Fukushima H, Mukawa T, Toyoda H, Wu LJ, Zhao MG, et al. Upregulation of CREB-mediated transcription enhances both short- and long-term memory. J Neurosci. 2011;31(24):8786-802.

5 Mamiya N, Fukushima H, Suzuki A, Matsuyama Z, Homma S, Frankland PW, et al. Brain region-specific gene expression activation required for reconsolidation and extinction of contextual fear memory. J Neurosci. 2009;29(2):402-13.

6 Hasegawa S, Fukushima H, Hosoda H, Serita T, Ishikawa R, Rokukawa T, et al. Hippocampal clock regulates memory retrieval via Dopamine and PKA-induced GluA1 phosphorylation. Nat Commun. 2019;10(1):5766.

7 Takahashi S, Fukushima H, Yu Z, Tomita H, Kida S. Tumor necrosis factor α negatively regulates the retrieval and reconsolidation of hippocampus-dependent memory. Brain Behav Immun. 2021;94:79-88.

8 Anagnostaras SG, Josselyn SA, Frankland PW, Silva AJ. Computer-assisted behavioral assessment of Pavlovian fear conditioning in mice. Learn Mem. 2000;7(1):58-72.

9 Fukushima H, Zhang Y, Archbold G, Ishikawa R, Nader K, Kida S. Enhancement of fear memory by retrieval through reconsolidation. Elife. 2014;3:e02736.

10 Fukushima H, Zhang Y, Kida S. Interactions between the amygdala and medial prefrontal cortex as upstream regulators of the hippocampus to reconsolidate and enhance retrieved inhibitory avoidance memory. Mol Brain. 2021;14(1):44.

11 Fukushima H, Zhang Y, Kida S. Active Transition of Fear Memory Phase from Reconsolidation to Extinction through ERK-Mediated Prevention of Reconsolidation. J Neurosci. 2021;41(6):1288-300.

12 Zhang Y, Fukushima H, Kida S. Induction and requirement of gene expression in the anterior cingulate cortex and medial prefrontal cortex for the consolidation of inhibitory avoidance memory. Mol Brain. 2011;4:4.

13 Stierl M, Stumpf P, Udwari D, Gueta R, Hagedorn R, Losi A, et al. Light modulation of cellular cAMP by a small bacterial photoactivated adenylyl cyclase, bPAC, of the soil bacterium Beggiatoa. J Biol Chem. 2011;286(2):1181-8.

14 Stierl M, Penzkofer A, Kennis JT, Hegemann P, Mathes T. Key residues for the light regulation of the blue light-activated adenylyl cyclase from Beggiatoa sp. Biochemistry. 2014;53(31):5121-30.

15 Luyben TT, Rai J, Li H, Georgiou J, Avila A, Zhen M, et al. Optogenetic Manipulation of Postsynaptic cAMP Using a Novel Transgenic Mouse Line Enables Synaptic Plasticity and Enhances Depolarization Following Tetanic Stimulation in the Hippocampal Dentate Gyrus. Front Neural Circuits. 2020;14:24.

16 Gasser C, Taiber S, Yeh CM, Wittig CH, Hegemann P, Ryu S, et al. Engineering of a red-light-activated human cAMP/cGMP-specific phosphodiesterase. Proc Natl Acad Sci U S A. 2014;111(24):8803-8.

17 Franklin KB, Paxinos G. The mouse brain in stereotaxic coordinates*.* Elsevier Academic: San Diego; 1997.

18 Ishikawa R, Kim R, Namba T, Kohsaka S, Uchino S, Kida S. Time-dependent enhancement of hippocampus-dependent memory after treatment with memantine: Implications for enhanced hippocampal adult neurogenesis. Hippocampus. 2014;24(7):784-93.

19 Tsuji R, Inoue H, Uehara M, Kida S. Dietary magnesium deficiency induces the expression of neuroinflammation-related genes in mouse brain. Neuropsychopharmacol Rep. 2021;41(2):230-36.

20 Parasuraman S, Raveendran R, Kesavan R. Blood sample collection in small laboratory animals. J Pharmacol Pharmacother. 2010;1(2):87-93.

21 Kida S, Josselyn SA, Peña de Ortiz S, Kogan JH, Chevere I, Masushige S, et al. CREB required for the stability of new and reactivated fear memories. Nat Neurosci. 2002;5(4):348-55.

22 Hori H, Yoshida F, Itoh M, Lin M, Niwa M, Ino K, et al. Proinflammatory status-stratified blood transcriptome profiling of civilian women with PTSD. Psychoneuroendocrinology. 2020;111:104491.

23 Foa E. *PDS: Posttraumatic Stress Diagnostic Scale: Manual* . *.* Pearson: Minneapolis; 1995.

24 Itoh M, Ujiie Y, Nagae N, Niwa M, Kamo T, Lin M, et al. The Japanese version of the Posttraumatic Diagnostic Scale: Validity in participants with and without traumatic experiences. Asian J Psychiatr. 2017;25:1-5.

25 Blake DD, Weathers FW, Nagy LM, Kaloupek DG, Gusman FD, Charney DS, et al. The development of a Clinician-Administered PTSD Scale. J Trauma Stress. 1995;8(1):75-90.

26 Sheehan DV, Lecrubier Y, Sheehan KH, Amorim P, Janavs J, Weiller E, et al. The Mini-International Neuropsychiatric Interview (M.I.N.I.): the development and validation of a structured diagnostic psychiatric interview for DSM-IV and ICD-10. J Clin Psychiatry. 1998;59 Suppl 20:22-33;quiz 34-57.

27 Asukai N, Kato H, Kawamura N, Kim Y, Yamamoto K, Kishimoto J, et al. Reliability and validity of the Japanese-language version of the impact of event scale-revised (IES-R-J): four studies of different traumatic events. J Nerv Ment Dis. 2002;190(3):175-82.

28 Weiss D, Marmar C. The Impact of Event Scale-Revised. In: Wilson JP KT, editor *Assessing Psychological Trauma and PTSD: A Practitioner"s Handbook*  New York: Guilford Press; 1997. p. 399–411.

29 Nakazato K, Mizuguchi T. Development and validation of Japanese version of State-Trait Anxiety Inventory. Japanese J Psychosom Intern Med. 1982;22:107–12.

30 Spielberger CD, Gorsuch RL, Lushene RE. Manual for the state-trait anxiety inventory. Consulting Psychologist Press: Palo Alto.; 1970.

31 Kojima M, Furukawa TA, Takahashi H, Kawai M, Nagaya T, Tokudome S. Cross-cultural validation of the Beck Depression Inventory-II in Japan. Psychiatry Res. 2002;110(3):291-9.

32 Beck AT, Steer RA, Brown GK. BDI-II, Beck Depression Inventory: manual. *.* Psychological Corp: San Antonio, TX.; 1996.

33 Huang dW, Sherman BT, Lempicki RA. Systematic and integrative analysis of large gene lists using DAVID bioinformatics resources. Nat Protoc. 2009;4(1):44-57.

34 Muthén LK, Muthén B. *Mplus user’s guide. 7th ed.*  *.* Muthén & Muthén: Los Angeles; 2012.

**
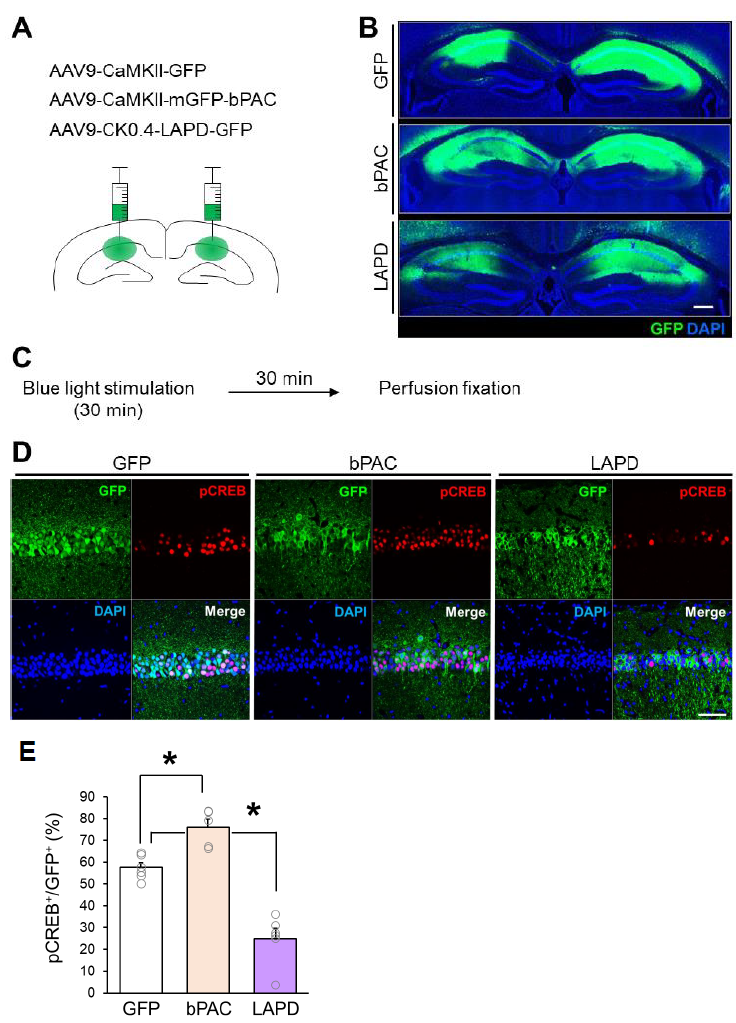
Supplementary Figure**

**Supplementary Fig. 1**

Phosphorylation levels of CREB in the dorsal hippocampus after optogenetic manipulation of cAMP levels by bPAC and LAPD.

**A**, Schematic illustration of virus injection. **B**, mGFP expression in the dorsal hippocampus. Scale bar, 300 μm. **C**, Experimental design. **D**, Representative immunohistochemical staining of GFP^+^, pCREB^+^, DAPI^+^ and merged cells in the CA1 region of the dorsal hippocampus. Scale bar, 100 μm. **E**, pCREB levels in the CA1 region of the dorsal hippocampus. GFP, n = 6; bPAC, n = 5; LAPD, n = 6. **p* < 0.05, *post hoc* Bonferroni's test. Error bars indicate SEM.


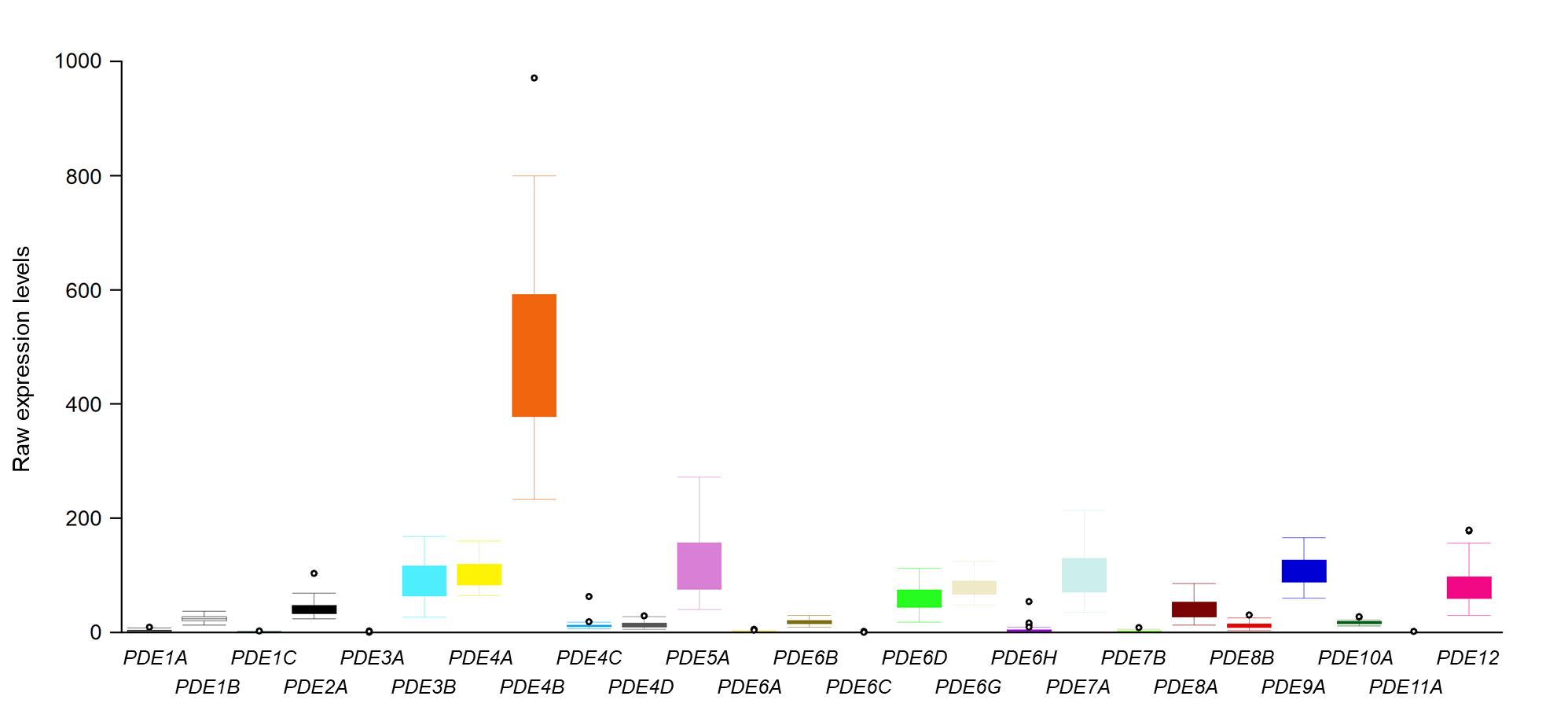


**Supplementary Fig. 2**

Microarray-based raw expression levels of PDE family genes in the blood of humans (patients and controls are combined).
